# A RNA-Seq Analysis of the Response of Photosynthetic System to Low Nitrogen Supply in Maize Leaf

**DOI:** 10.3390/ijms18122624

**Published:** 2017-12-05

**Authors:** Xiaohuan Mu, Qinwu Chen, Fanjun Chen, Lixing Yuan, Guohua Mi

**Affiliations:** Department of Plant Nutrition, China Agricultural University, Beijing 100193, China; muxiaohuan@cau.edu.cn (X.M.); chenqw@cau.edu.cn (Q.C.); caucfj@cau.edu.cn (F.C.); yuanlixing@cau.edu.cn (L.Y.)

**Keywords:** chlorophyll fluorescence, electron transport rate, nitrogen, photosynthesis, PSI, PSII, maize

## Abstract

Nitrogen is a major limiting factor for crop productivity. The relationship between photosynthesis and nitrogen nutrition has been widely studied. However, the molecular response of leaf photosynthesis to low nitrogen supply in crops is less clear. In this study, RNA sequencing technology (RNA-Seq) was used to investigate the gene expressions related to photosynthesis in maize in response to low nitrogen supply. It was found that low nitrogen supply down-regulated the expression of genes involved in photosystem I (PSI) and photosystem II (PSII). Thus, low nitrogen supply down-regulated the expression of genes related to the antenna system, reduced light absorption, light transport, and electron transport. Correspondingly, the parameters related to chlorophyll fluorescence were very sensitive to nitrogen deficiency. Under low nitrogen supply, leaf chlorophyll content, actual quantum yield of PSII photochemistry, photochemical quenching, and electron transport rate, were reduced. However, the thermal diffusion and chlorophyll fluorescence were increased. RNA-Seq was used to analyze the genes involved in the response of leaf photosynthesis to low nitrogen supply in maize. These results highlight the possibility of utilizing chlorophyll fluorescence parameters, and the related genes, as indicators for plant nitrogen nutrition. This could lead to the development of new tools to make precise nitrogen fertilizer recommendations and select nitrogen-efficient genotypes.

## 1. Introduction

Nitrogen (N) is a fundamental constituent of many cell components such as amino acids, proteins, cell walls, membranes and nucleic acids. Nitrogen deficiency reduces plant growth and development, photosynthesis, leaf area, and ultimately limits plant productivity [1,2,3,4]. For a sustainable crop production system, there is a requirement to reduce nitrogen fertilizer input, and increase nitrogen use efficiency. This may be achieved by understanding the relationship between N nutrition and the photosynthetic rate in the leaf [5,6].

Photosynthesis is a biological process whereby the sun’s energy is captured and stored in a series of events that convert the pure energy of light into the biochemical energy needed to power life [7]. Photosynthesis includes two reactions: light reactions and carbon reduction reactions [7]. Light reactions take place on thylakoid membranes. Thylakoid membranes mainly include four membrane protein complexes: photosystem I (PSI), photosystem II (PSII), cytochrome *b*_6_*f* complex (Cyt *b*_6_*f*), and adenosine triphosphate (ATP) synthase. Carbon reduction reactions take place in the stroma of chloroplast. Ribulose-1,5-bisphosphate carboxylase (Rubisco), phosphoenolpyruvate carboxylase (PEPc) and pyruvate orthophosphate dikinase (PPDK) are the key enzymes for C_4_ plants. Light is absorbed by pigments (mainly chlorophylls) associated with two photosystems: PSI and PSII. For light energy to be stored by photosynthesis, it is channeled by a number of different processes including photochemistry, heat dissipation (including photo-protective heat dissipation and other heat dissipation), and chlorophyll fluorescence [8]. Chlorophyll fluorescence is an important parameter in representing changes in the growth environment and function of the photosynthetic process [8]. For example, in rice, nitrogen deficiency decreases the actual quantum yield of PSII photochemistry (Φ_PSII_), the maximal efficiency of PSII photochemistry (*F*v/*F*m), excitation energy capture efficiency of PSII (*F*v’*/F*m’), as well as the electron transport rate (ETR) [9].

Photosynthesis depends on many physiological and biochemical processes such as stomatal conductance, intercellular CO_2_ concentration, photochemical capacity of PSII, and contents and activities of carbon fixation enzymes [10]. Photosynthesis has a positive relationship with leaf nitrogen [3,5,11,12]. This is because about 70% of leaf nitrogen is located in the chloroplast [11,13]. Nitrogen deficiency reduces the content of chlorophyll, Cyt *f*, coupling factor, N content of thylakoid in light reactions, as well as the ETR [5,14]. Nitrogen deficiency also decreased the content and/or activity of Rubisco, PEPc and PPDK [3,5,11,12]. The decreased photosynthesis in nitrogen deficiency may be caused by different reasons in different plants under different conditions. For example, decreased photosynthesis in low nitrogen was mainly associated with lower stomatal conductance in sorghum under outdoor pot-culture conditions [10]. At light saturation, the decline in photosynthesis in nitrogen deficiency was mainly caused by the limitation of reduced mesophyllic activity, rather than by stomatal limitation in sunflowers [15]. Nitrogen deficiency repressed ΦPSII, causing decreased ETR to match the decreased requirements for ATP and triphosphopyridine nucleotide (NADPH) in the case of decreased CO_2_ assimilation capacity in maize grown outdoors [16].

Maize is an important crop worldwide with multiple purposes such as food, fodder, and bioenergy. Although the relationship between nitrogen deficiency and photosynthesis has been widely studied in maize, less is known about the molecular mechanism underlying the response of the photosynthetic system in maize to nitrogen deficiency. Such information is important not only for improving photosynthesis performance under insufficient nitrogen supply via gene manipulation, but also for the development of potential molecular tools for the diagnosis of plant nitrogen nutrition status. This could be used in both the selection of nitrogen-efficient genotypes and precision nitrogen fertilizer management. In the present study, using an integrated approach including physiological analysis and RNA sequencing (RNA-Seq), we aimed to explore the genes related to photosynthesis under nitrogen deficiency, especially those related to light reactions. 

## 2. Results

### 2.1. Effect of Nitrogen Supply on Biomass 

Nitrogen supply has a significant effect on biomass and nitrogen accumulation. Low nitrogen treatment reduced total biomass by 15%, and shoot biomass by 25% (Table 1). However, low nitrogen treatment increased the root–shoot ratio by about two-thirds compared with the high nitrogen treatment, indicating that nitrogen-starved plants allocated more biomass to the root. Total nitrogen accumulation, as well as shoot nitrogen accumulation, in low nitrogen plants were reduced by 74% and 77%, respectively (Table 1). 

### 2.2. Effect of Nitrogen Supply on Leaf Photosynthesis

**Figure 1:**
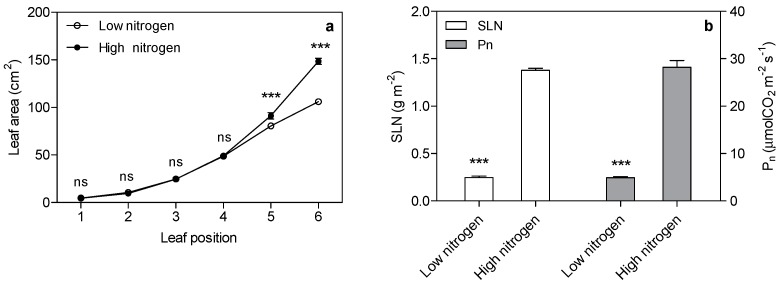
Effect of nitrogen supply on leaf area (**a**), specific leaf nitrogen (SLN) and photosynthetic rate (*p*_n_) of the sixth leaf in maize (**b**). Bars denote the standard error (SE) of the mean. ns: not significant (*p* > 0.05); ***: significant at *p* < 0.001.

**Figure 2:**
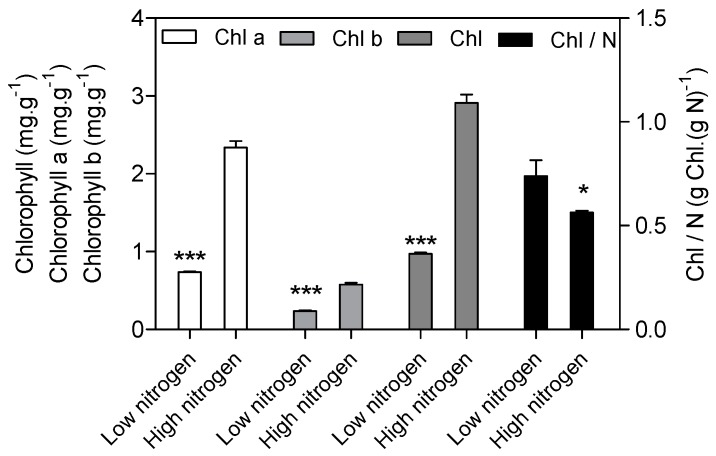
Effect of nitrogen supply on chlorophyll (Chl) concentration and Chl/N in the sixth leaf of maize. High nitrogen: 4 mM Ca(NO_3_)_2_; Low nitrogen: 40 µM Ca(NO_3_)_2_. Bars denote the SE of the mean. ns: not significant (*p* > 0.05); *, ** and ***: significant at *p* < 0.05, 0.01 and 0.001, respectively.

As expected, low nitrogen significantly reduced the leaf area (Figure 1a). Nitrogen supply has a significant effect on specific leaf nitrogen (SLN) and the photosynthetic rate (Figure 1b). SLN is lower (82%) in low nitrogen treatment compared with high nitrogen treatment. The photosynthetic rate in low nitrogen stress is reduced by 83% compared with high nitrogen treatment. The chlorophyll (Chl), chlorophyll a, and chlorophyll b concentration are lower (67%, 69% and 59% respectively) in low nitrogen plants compared with high nitrogen plants (Figure 2). The ratio of Chl to N was 31% higher in low nitrogen treatment compared with high nitrogen treatment.

### 2.3. Low Nitrogen-Induced Gene Expression in the Leaf

We used RNA-Seq analysis of the mature tissue of an expanding leaf in order to unravel the molecular mechanisms underlying the response of the photosynthetic system to low nitrogen supply. A total of more than 33 million high quality 125-bp paired-end reads were generated by RNA-Seq for each treatment. Comparisons of two biological replicates showed that expression values were highly correlated (average *R*^2^ = 0.9288, Figure S1). 

**Figure 3:**
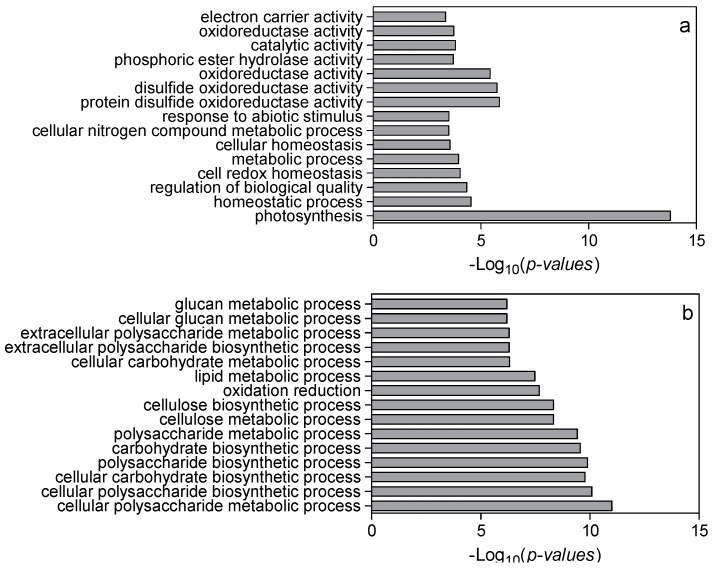
Significantly enriched gene ontology (GO) terms in downregulated (**a**) and upregulated (**b**) differentially expressed genes in low nitrogen versus high nitrogen in the sixth leaf. High nitrogen: 4 mM Ca(NO_3_)_2_; low nitrogen: 40 µM Ca(NO_3_)_2_. After GO analysis, every significantly enriched GO term has a *p*-value. The smaller *p*-value, the more reliable GO result is.

**Figure 4:**
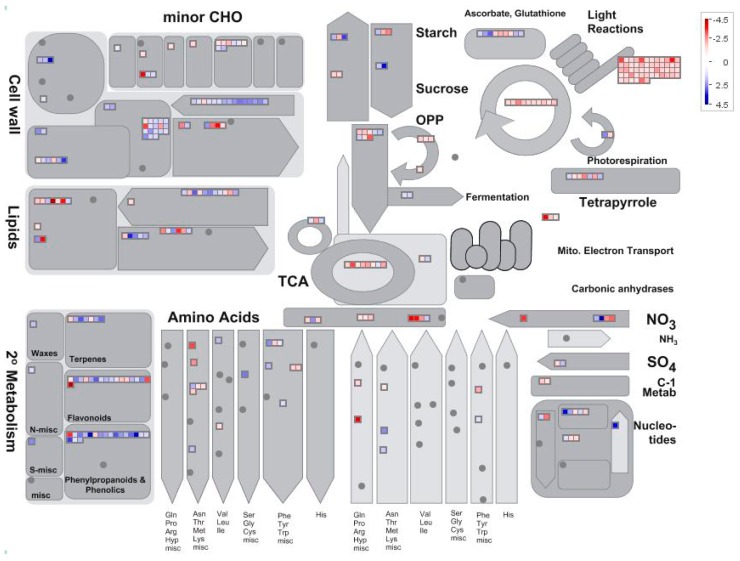
The distribution of different gene expressions involved in metabolism in maize leaf (low nitrogen versus high nitrogen). High nitrogen: 4 mM Ca(NO_3_)_2_; Low nitrogen: 40 µM Ca(NO_3_)_2_.

Setting thresholds of a False Discovery Rate of less than 0.05 and a two-fold change in expression, we found 1625 differentially expressed genes (DEGs) in low nitrogen compared with high nitrogen treatment. Of these genes, 928 were downregulated whilst 697 upregulated (Supplemental Table S1). The identified genes were subjected to gene ontology term (GO) enrichment analysis. The downregulated DEGs were mainly involved in photosynthesis, homeostatic processes, regulation of nitrogen metabolism, redox activity, and response to abiotic stimulus (Figure 3a; Supplemental Table S2). The upregulated DEGs were mainly involved in cellular polysaccharide processes, carbohydrate biosynthesis processes and cellular metabolic processes (Figure 3b; Supplemental Table S2). Using MapMan analysis, it is further identified that most downregulated DEGs were involved in photosynthesis processes, TCA cycling, and nitrogen assimilation processes (Figure 4; Supplemental Table S3). The upregulated DEGs were mainly associated with lipids, the cell wall, and secondary metabolism processes (Figure 4; Supplemental Table S3). 

**Figure 5:**
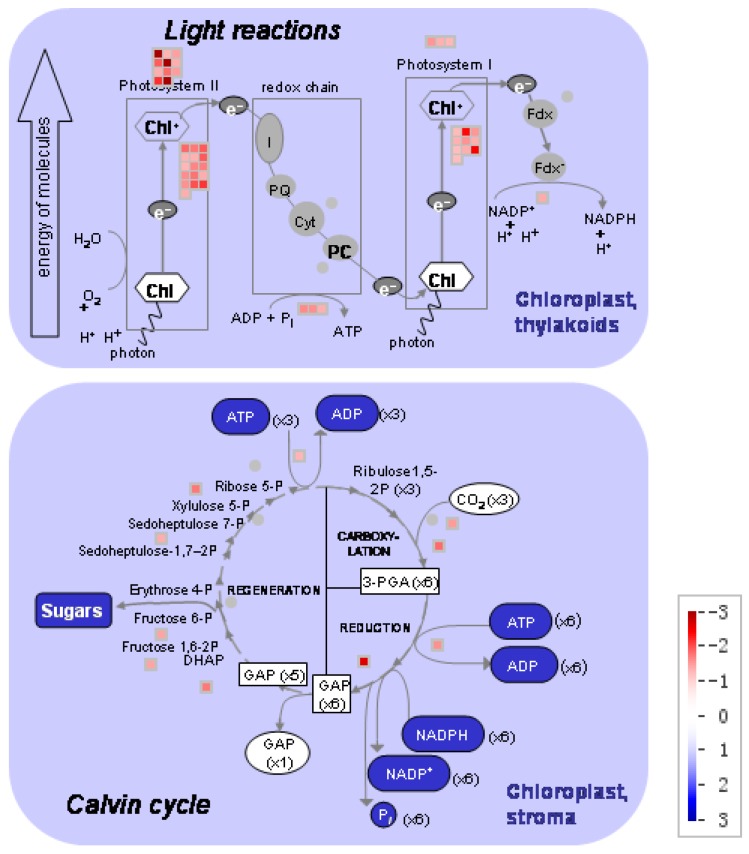
The distribution of different gene expressions related to photosynthesis in the maize leaf (low nitrogen versus high nitrogen). High nitrogen: 4 mM Ca(NO_3_)_2_; Low nitrogen: 40 µM Ca(NO_3_)_2_.

The DEGs involved in photosynthesis were analyzed (Figure 5; Supplemental Table S4). Under low nitrogen supply, 11 DEGs encoding light harvesting complex II (LHCII) and three DEGs encoding LHCI were downregulated, 16 DEGs encoding PSII polypeptide subunits and 10 DEGs encoding PSI polypeptide subunits were downregulated, 3 DEGs encoding ATP synthase were downregulated, 1 DEG encoding ferredoxin reductase was downregulated, and 10 DEGs involved in Calvin cycle were downregulated. In those DEGs, GRMZM2G351977, GRMZM2G414192, GRMZM2G149428 and GRMZM2G092427 encoded components of PSII: *Lhcb1*, *Lhcb2*, *Lhcb5*, respectively. The genes GRMZM2G038519 and GRMZM2G072280 encoded components of PSI: *Lhca1* and *Lhca2*, respectively.

### 2.4. Effect of Low Nitrogen Supply on Leaf Chlorophyll Fluorescence

**Figure 6:**
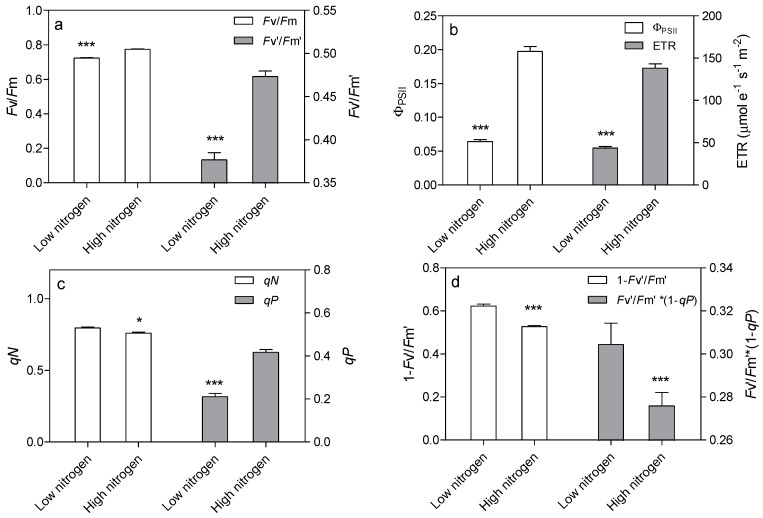
Effect of nitrogen supply on chlorophyll fluorescence parameters in the sixth leaf of maize. Effect of nitrogen supply on *F*v/*F*m and *F*v’/*F*m’ (**a**), on Φ_PSII_ and ETR (**b**), on *qN* and *qP* (**c**), on 1 − *F*v’/*F*m’ and *F*v’/*F*m’·* (1 − *qP*) (**d**). High nitrogen: 4 mM Ca(NO_3_)_2_; Low nitrogen: 40 µM Ca(NO_3_)_2_. Bars denote the SE of the mean. ns: not significant (*p* > 0.05); *, ** and ***: significant at *p* < 0.05, 0.01 and 0.001, respectively.

Leaf chlorophyll fluorescence was analyzed to confirm the effect of low nitrogen supply on the light reaction system. Nitrogen supply has a great effect on chlorophyll fluorescence (Figure 6). The *F*v/*F*m and *F*v’/*F*m’ were 6.6% and 20.4% lower in low-nitrogen plants compared with high nitrogen plants, respectively (Figure 6a). The actual quantum yield of PSII photochemistry (Φ_PSII_) was reduced by 67.4% in low nitrogen treatment compared to high nitrogen treatment (Figure 6b). The electron transport rate (ETR) was 68.3% lower in low nitrogen plants than in high nitrogen plants (Figure 6b). The non-photochemical quenching (*qN*) was 4.6% higher in low nitrogen treatment than in high nitrogen treatment (Figure 6c). The photochemical quenching (*qP*) was lower (49.6%) in low nitrogen treatment than in high nitrogen treatment. The 1 − *F*v’/*F*m’ and *F*v’/*F*m’·* (1*qP*) were higher (18.3% and 10.3% respectively) in low nitrogen plants compared to high nitrogen plants (Figure 6d). 

## 3. Discussion

Increased photosynthesis with less input of land, water, nutrients, etc., is essential to sustainably meet global food and bioenergy demands [17,18]. New models have been proposed to increase the efficiency of light capture, light energy conversion, and carbon capture and conversion, possibly by rapidly developing genetic engineering technologies [18]. Photosynthesis has a close relationship with leaf nitrogen. In our study, leaf area was reduced by 22% in low nitrogen treatment. The photosynthetic rate in low nitrogen plants was 83% lower than in high nitrogen plants. Thus, the decrease in photosynthesis is the main reason for the decreasing biomass. 

Nitrogen is a constituent of chlorophyll, photosynthetic enzymes (included Rubisco, PEPc and PPDK), and thylakoid membranes. These cellular features are located in chloroplast. About three-quarters of total nitrogen is found in chloroplast [11,13]. The optimal SLN for the maximum photosynthetic rate (*p*_n_) is reported to be approximately 1.5 g·m^−2^ in field-grown maize [4,19,20,21]. In this research, the specific leaf nitrogen is only 0.25 g·m^−2^ in low nitrogen plants, 82% lower than that of the control treatment. This correlates well with the 83% reduction in the photosynthetic rate. This reduction in SLN is the result of the contribution of the different photosynthetic components mentioned above [3,5,11]. Here, we focused on the effect of nitrogen deficiency on the light reaction in photosynthesis. 

Complexes PSII and PSI take part in light absorption, transport, and conversion. Photosystems are composed of two sections: (1) a reaction center devoted to the conversion of light energy into chemical energy; and (2) an antenna system that increases the capacity of light absorption and contributes to photoprotection [22]. The antenna system consists of many light-harvesting complexes (*Lhc*). Different members are associated with PSI (*Lhca* proteins) and PSII (*Lhcb* proteins) [23]. Over 60% of all the chlorophyll in plants is bound to light-harvesting complexes. Nitrogen deficiency leads to reduced total chlorophyll, chlorophyll a, and chlorophyll b contents (Figure 2) [3,5,11]. The ratio of chlorophyll to leaf N could represent N allocation into chlorophyll [24]. The ratio of chlorophyll to leaf N was higher in low nitrogen treatment (Figure 2). This suggests that maize tends to invest relatively more N into light harvesting complexes (LHCs), PSI and PSII under nitrogen stress. Chlorophyll fluorescence is frequently used to monitor the responses of the photosynthetic apparatus to environmental stressors [8,25] including temperature [26], nitrogen [16] and drought [27]. In our study, all of the chlorophyll fluorescence parameters were affected by nitrogen supply. The ETR was 68% lower in low nitrogen treatment compared with high nitrogen treatment (Figure 6b), suggesting that low nitrogen impairs electron transport systems. Nitrogen stress reduced by 6.6% in *F*v/*F*m and 20.4% in *F*v’/*F*m’ (Figure 6a), suggesting that an energy dissipation exists in low nitrogen plants [28]. Light energy absorbed by chlorophyll in a leaf has three fates: it can be used to drive photosynthesis (photochemistry), excess energy can be dissipated as heat, or it can be re-emitted as light-chlorophyll fluorescence [8]. The fraction of light absorbed in PSII that is dissipated thermally (D = 1 − *F*v’/*F*m’) was 18.3% higher in low nitrogen plants than in high nitrogen plants (Figure 6d). Accordingly, nitrogen-deficient plants had a higher non-photochemical quenching (*qN*) in low nitrogen treatment (Figure 6c) [9,16]. The fraction of light absorbed in PSII that is utilized in PSII photochemistry could be represented by Φ_PSII_. Φ_PSII_ was 67.4% lower in low nitrogen plants compared with in high nitrogen plants. Nitrogen-deficient plants had a lower photochemical quenching (*qP*) in low nitrogen treatment (Figure 6c). Lower *qP* means a lower degree of open PSII, resulting in lower Φ_PSII_. The fraction of light absorbed in PSII that is dissipated in chlorophyll fluorescence (*F*v’/*F*m · * (1 − *qP*)) is increased by 10.3% in low nitrogen plants compared with high nitrogen plants (Figure 6d). Thus, it is supposed that low nitrogen impaired PSII, as well as electron transport. Excess energy can be dissipated thermally or re-emitted as light-chlorophyll fluorescence. In accordance, RNA-Seq analysis found that about 27 DEGs encoding PSII systems, and 13 DEGs encoding PSI systems, were downregulated by low nitrogen supply (Figure 5). Among these downregulated DEGs, GRMZM2G351977, GRMZM2G414192, GRMZM2G149428 and GRMZM2G092427 encoded *Lhcb1*, *Lhcb2*, *Lhcb5*, and *Lhcb6*, respectively. Six different proteins comprise the antenna system of PSII: three minor proteins (CP29, CP26 and CP24) encoded by *Lhcb4*, *Lhcb5* and *Lhcb6*; and the major complex LHCII, which consists of three proteins encoded by *Lhcb1*, *Lhcb2* and *Lhcb3*, of which the *Lhcb1* and *Lhcb2* proteins are by far the most abundant [29]. In antisense plants of *Arabidopsis thaliana*, there is an absence of the *Lhcb1* and *Lhcb2* proteins, accompanied by reduced light absorption [29]. In *Arabidopsis thaliana*, mutant lines of *Lhcb5* and *Lhcb6* have a decreased efficiency of energy transfer from LHCII to the reaction center of PSII [30]. In addition, *Lhcb6* absence limits plastoquinone diffusion, and thus inhibits the electron transport rate [30]. Thus, lower expression of GRMZM2G351977, GRMZM2G414192, GRMZM2G149428 and GRMZM2G092427 resulted in lower synthesis of *Lhcb1*, *Lhcb2*, *Lhcb5*, and *Lhcb6*, respectively. Reduced expression of these genes also resulted in limited light absorption, light transport and electron transport. This is consistent with the results of chlorophyll fluorescence.

GRMZM2G038519 and GRMZM2G072280 encoded *Lhca1* and *Lhca2*, respectively. The antenna systems of PSI exist as *Lhca1*/*4* and *Lhca2*/*3* dimers. The absence of one antenna complex leaves a “hole” in the structure that cannot be filled by other *Lhca* proteins [31]. Therefore, lower expression of GRMZM2G038519 and GRMZM2G072280 resulted in lower synthesis of antenna systems of PSI, thus reducing light absorption in low nitrogen stress.

Overapplication of nitrogen has been a common problem in China, resulting in low N use efficiency (NUE) and environmental pollution [32]. NUE can be improved by precision nitrogen management and nitrogen-efficient cultivars. For both approaches, it is necessary to diagnose plant nitrogen nutrition status timely and precisely. Chlorophyll fluorescence has been used to estimate plant nitrogen status [33]. The findings of this research suggest that chlorophyll fluorescence and the related genes could potentially be explored for developing new tools for the diagnosis of plant nutrition status. Based on the chlorophyll fluorescence parameters, and the differentially expressed genes in response to nitrogen deficiency, nitrogen-efficient genotypes could be discerned at the early seedling stage. Nitrogen deficiency could be detected at the early seedling stage so that nitrogen application recommendation can be made in a timely manner. Indeed, in potatoes, it was found that chlorophyll fluorescence can discriminate between genotypes, predict plant age, and yield performance under field conditions [34]. 

In conclusion, our results show that low nitrogen supply results in the downregulation of key genes involved in light reactions of photosynthesis. Of particular importance are PSI and PSII genes including GRMZM2G351977, GRMZM2G414192, GRMZM2G149428, GRMZM2G092427, GRMZM2G038519 and GRMZM2G072280. Downregulated expression of those genes resulted in reduced light absorption and light transport. As a result, the parameters relating to chlorophyll fluorescence (Φ_PSII_, electron transport rate, *qP*) were lower, which contributed to a lower photosynthetic rate. These findings suggest the potential to utilize chlorophyll fluorescence parameters, as well as the related genes, as indicators of plant nitrogen nutrition. In addition, this could be used to develop new tools to make precise nitrogen fertilizer recommendations, and select nitrogen-efficient genotypes.

## 4. Materials and Methods

### 4.1. Plant Material and Growth Condition

Seeds of maize (*Zea mays* L.) inbred line B73 were sterilized in 10% (*v*/*v*) H_2_O_2_ for 30 min, washed with distilled water, and then soaked in saturated CaSO_4_ solution for 8 h. Then, the seeds were washed and placed between sheets of filter paper and germinated in the dark at room temperature. When the roots were approximately 1 cm long, uniform seedlings were wrapped in filter paper and transferred into a plastic container filled with distilled water. The seedlings with two visible leaves were then transferred into porcelain pots (4 seedlings per pot) containing 2 L of nutrient solution after the endosperm was removed. The plants were grown in a growth chamber at 28/22 °C with a 14/10 h light/dark cycle. During the light cycle, the photosynthetic photon flux density was 250–300 µmol·m^−2^·s^−1^ at canopy height.

The nutrient solution contained 0.75 mM K_2_SO_4_, 0.1 mM KCl, 0.25 mM KH_2_PO_4_, 0.65 mM MgSO_4_, 0.13 mM Ethylenediaminetetraacetic acid (EDTA)-Fe, 1.0 µM MnSO_4_, 1.0 µM ZnSO_4_, 0.1 µM CuSO_4_ and 0.005 µM (NH_4_)_6_Mo_7_O_24_ [35]. Plants were supplied with half-strength nutrient solution containing 4.0 mM NO_3_ (provided as Ca(NO_3_)_2_) for 2 days, and then transferred into full-strength solution with 4.0 mM NO_3_ (high nitrogen). When the third leaf was fully expanded (approximately 6 days later), half of the plants were moved into a solution with 40 µM NO_3_ (low nitrogen), with CaCl_2_ added to equalize calcium concentration between the treatments. The pH of the solution was adjusted to 5.8–6.0 with KOH. The solution was renewed every other day and was aerated continuously by a pump. The pots were arbitrarily placed and rotated when the nutrient solution was renewed.

### 4.2. Plant Weight, Leaf Area, and Photosynthesis

When the sixth leaf was fully expanded, plants were harvested and separated into root, leaf 6, and shoot (other leaves plus stems). The length and width of the leaves were measured with a ruler. Half of sixth leaves were stored at −80 °C. Other samples were dried in an oven at 70 °C. Dried samples were weighed and ground to a powder and the nitrogen concentration was determined by an elemental analyzer (vario MACRO, Elementar, Langenselbold, Germany). 

The sixth expanded leaf from 16 plants of each nitrogen treatment was used to measure the net photosynthetic rate by LI6400 (LI-COR, Lincoln, NE, USA) at a light density of 800 µmol·m^−2^·s^−1^ [5]. The CO_2_ concentration inside the chamber was controlled at 400 ± 1 µmol CO_2_ (mol air)^−1^. Chlorophyll (Chl) was extracted using acetone and ethanol, and the absorbance of extracts was spectrophotometrically measured at 645 and 663 nm [36].

### 4.3. Chlorophyll Fluorescence

Chlorophyll fluorescence was measured with an integrating fluorescence fluorometer (LI-6400, Leaf chamber fluorometer, LI-COR, Lincoln, NE, USA). The fluorescence instable state (*F*s), maximum fluorescence under light (*F*m’) and minimum fluorescence (*F*o’) were measured under light density of 800 µmol·m^−2^·s^−1^ and 400 ± 1 µmol CO_2_ (mol air)^−1^. After dark-adaptation of samples for 1 h, the minimal fluorescence (*F*o) and maximum fluorescence (*F*m) in darkness were measured. Other fluorescent parameters were calculated as follows [37]: maximal photochemical efficiency (*F*v/*F*m):*F*v/*F*m = (*F*m − *F*o)/*F*m; excitation energy capture efficiency of PSII reaction centers (*F*v’/*F*m’):*F*v’/*F*m’ = (*F*m’ − *F*o’)/*F*m’; Φ_PSII_ = (*F*m’ − *F*s)/*F*m’; ETR = PPFD × Φ_PSII_ × 0.85 × 0.5 (where PPFD is photosynthetic photon flux density); Photochemical quenching (*qP*): *qP* = (*F*m’ − *F*s)/(*F*m’ − *F*o’); Non-photochemical quenching (*qN*): *qN* = (*F*m − *F*m’)/(*F*m − *F*o’). The following three derived chlorophyll fluorescence parameters were employed to analyze the allocation of fraction of excitation energy: D = 1 − *F*v’/*F*m’ is the fraction of photon energy absorbed in PSII and dissipated via thermal energy in the antenna; Φ_PSII_ represents the fraction of photon energy absorbed in PSII utilized for photosynthetic electron transport; Ex = *F*v’/*F*m’ ·* (1 − *qP*) is the estimate of the fraction of excess excitation energy re-emitted as light-chlorophyll fluorescence.

### 4.4. RNA Library Construction and Illumina Sequencing

The mature tissue of an expanding leaf was sampled. The samples were frozen in liquid nitrogen and stored at −80 °C. Total RNA was extracted [38]. Two replicates were used to RNA-Seq. RNA-Seq libraries were prepared according the manufacturer’s protocol of the Illumina Standard mRNA-Seq library preparation kit (Illumina, San Diego, CA, USA) and were sequenced to generate 125-nucleotide paired-end reads on an Illumina HiSeq platform (Illumina HisSeq 2500, San Diego, CA, USA). 

### 4.5. Bioinformatics Analysis of RNA-Seq Data

Raw reads were pre-processed to remove low quality regions and adapter sequences. At least 30 million clean reads were obtained. Clean reads from each sample were aligned to the maize reference genome (B73 RefGen_v3, available online: http://www.maizegdb.org/assembly/) using TopHat2 [39]. Aligned reads from TopHat2 mapping were subjected to String Tie for DeNovo transcript assembly [39,40]. The R package edgeR was used to identify the differentially expressed genes [41]. The expression of each gene was normalized to fragments per kilobase of transcript per million reads (FPKM) to compare different samples. Low-level expressed genes were removed and only genes with an expression level of at least 1 FPKM in at least two samples were kept for further analysis. A gene was regarded as differentially expressed if the false discovery rate (FDR) was less than 0.05, and had a log2 fold change higher than 1.

The Gene Ontology (GO) term enrichment of differential expressed genes was conducted using the web-based agriGO software (Available online: http://bioinfo.cau.edu.cn/agriGO/analysis.php). Singular enrichment analysis (SEA) was used to compute enriched categories by comparing a list of differentially expressed genes to all expressed genes. GO terms of gene sets of interest compared with the genome-wide background with an adjusted *p* value (FDR) cutoff of 0.01. MapMan was used to show the functional categorization of differentially expressed genes in different cellular and metabolic processes [42]. 

Raw sequencing data are stored at the Sequence Read Archive (Available online: http://www.ncbi.nlm.nih.gov/sra) under accession number GSE107562.

### 4.6. Statistical Analysis

Data were subjected to variance analysis using ANOVA procedure implemented in SPSS Statistics 19.0 (SPSS, Inc., Chicago, IL, USA). Differences were compared using the least significant difference test at 0.05 level of probability.

## Figures and Tables

**Table 1:** Effect of nitrogen supply on biomass and nitrogen accumulation in maize plants at the V6 stage.

N Treatment	Total Biomass	Shoot Biomass	Root-to-Shoot Ratio	Total N Content	Shoot N Content
	(g·plant^−1^)		(mg·plant^−1^)		
High Nitrogen	2.74 a	2.05 a	0.34 b	132.26 a	101.33 a
Low Nitrogen	2.32 b	1.54 b	0.51 a	33.89 b	23.48 b

Note: Different letters in the same column indicate significant differences between nitrogen treatments (*p* < 0.05).

## Supplementary Materials

Supplementary materials can be found at www.mdpi.com/1422-0067/18/12/2624/s1.
