# Supplementary material for: A RNA-Seq Analysis of the Response of Photosynthetic System to Low Nitrogen Supply in Maize Leaf
Source: Int J Mol Sci. 2017 Dec 5;18(12):2624. doi: 10.3390/ijms18122624 (PMC5751227; doi:10.3390/ijms18122624)
Supplement: Supplementary file 1 [file ijms-18-02624-s001.zip › Supplemental files/Support information.pdf]

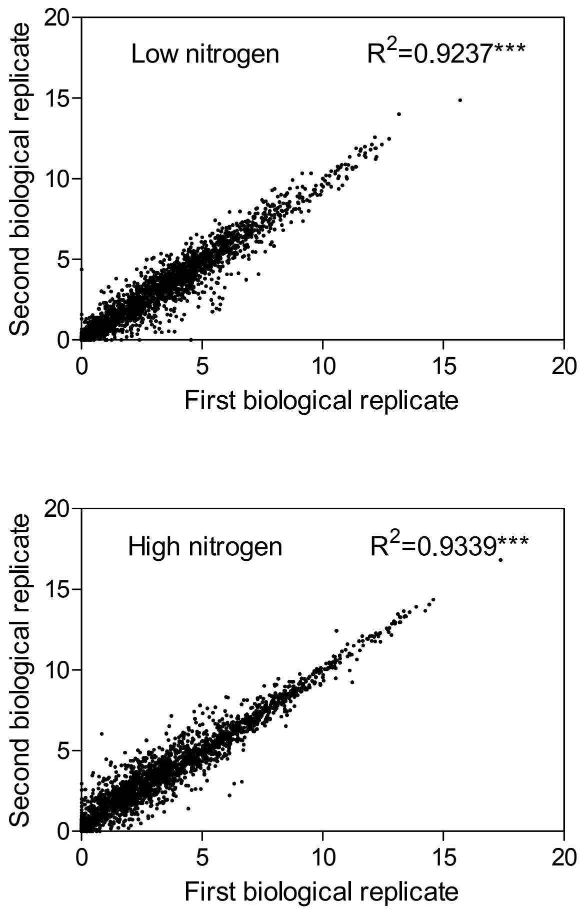

Figure 1S. Correlation between biological replicates. High nitrogen (HN): 4 mM $Ca(NO_3)_2$; Low nitrogen (LN) : 40 μM $Ca(NO_3)_2$.
